# Molecular Dynamics Simulations Show That Short Peptides Can Drive Synthetic Cell Division by Binding to the Inner Membrane Leaflet

**DOI:** 10.1021/acs.jpcb.4c04358

**Published:** 2024-09-03

**Authors:** Jan Steinkühler, Reinhard Lipowsky, Markus S. Miettinen

**Affiliations:** † Bio-Inspired Computation, Kiel University, Kaiserstraße 2, Kiel 24143, Germany; ‡ Kiel Nano, Surface and Interface Science KiNSIS, Kiel University, Christian-Albrechts-Platz 4, Kiel 24118, Germany; § Max Planck Institute of Colloids and Interfaces, Science Park Golm, Potsdam 14476, Germany; ∥ Department of Chemistry, University of Bergen, Bergen 5007, Norway; ⊥ Computational Biology Unit, Department of Informatics, University of Bergen, Bergen 5008, Norway

## Abstract

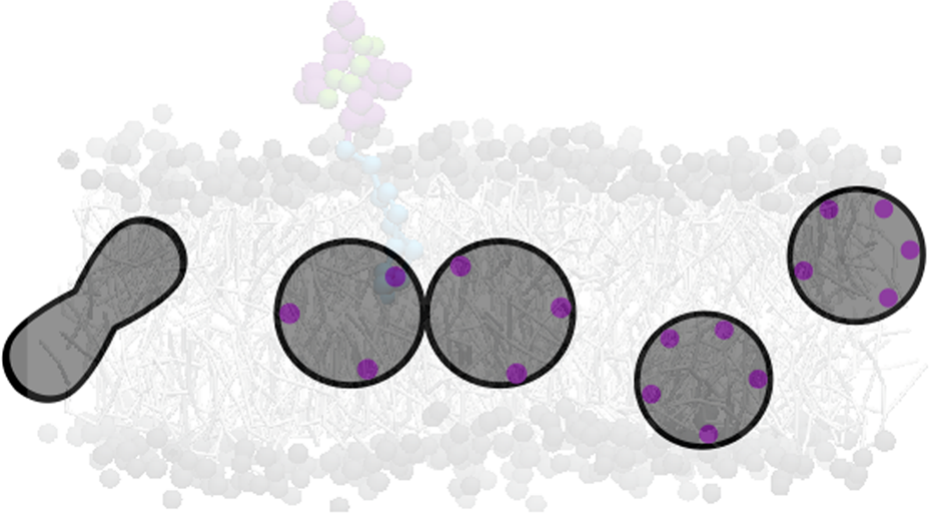

An important functionality of lifelike “synthetic cells” is to mimic cell division. Currently, specialized proteins that induce membrane fission in living cells are the primary candidates for dividing synthetic cells. However, interactions between lipid membranes and proteins that are not found in living cells may also be suitable. Here, we discuss the potential of short membrane-anchored peptides to induce cell division. Specifically, we used the coarse-grained MARTINI model to investigate the interaction between short membrane-anchored peptides and a lipid bilayer patch. The simulation revealed that the anchored peptide induces significant spontaneous curvature and suggests that the lipid–peptide complex can be considered as a conically shaped “bulky headgroup” lipid. By systematically increasing the electrostatic charge of the peptide, we find that membrane-anchored peptides may generate sufficiently large constriction forces even at dilute coverages. Finally, we show that when the peptide has an opposite charge to the membrane, the peptide may induce division by binding the inner membrane leaflet of a synthetic cell, that is, cell division from within.

## Introduction

The concept of “synthetic cells” refers to membrane-enclosed vesicles that exhibit lifelike features. The evolution, and proliferation, of synthetic cells requires a division mechanism. Mechanisms to induce division of lipid-membrane-bound vesicles have been known for a long time; however, in recent years, there has been a steep increase in approaches suitable for integration with other synthetic cell modules. Particularly interesting are approaches in which the division-inducing biomolecule is synthesized from a nucleic acid template. Therefore, proteins known to induce membrane fission at membrane necks of naturally occurring cells are promising candidates to reshape and divide lipid membranes outside of living cells.^1^ Indeed, proteins that induce the division of bacterial cells by polymerizing to membrane contracting filaments under GTP hydrolysis were shown to induce shape changes and sometimes division of lipid vesicles.^2,3^ However, large heterogeneities in the protein assemblies and unsuccessful division attempts within the same batch are common. The introduction of additional proteins from the MinDE family has been used successfully for better spatiotemporal control of filament assembly.^4^ Ultimately, by fine-tuning the protein interactions, this approach might provide a fully controlled division module. This would, however, be at the expense of energy input by GTP hydrolysis and a large burden on the synthetic cell protein synthesis machinery, which would have to synthesize multiple large and complex proteins. Furthermore a full division cycle would require the disassembly of division-inducing filaments from the membrane, requiring further energy input. Given these limitations, it is useful to notice that synthetic cells provide a new context compared to the evolved molecular interactions between proteins and membranes in cells; therefore, it is interesting to ask whether “noncanonical” interactions between membranes and proteins are also able to induce synthetic cell division. This might lead to simpler and easier control of division modules. Along these lines, the full division of vesicles by the combination of constriction proteins and DNA nanostars has been demonstrated,^5^ as well as by light-controlled lipid oxidation,^6^ or adsorbed green fluorescent proteins.^7^ In these works, synthetic cell division was accomplished by a constriction force that acts on membrane necks and arises from the elastic forces within an asymmetric membrane.^7,8^ Until now, the experimental demonstrations have had the serious limitation that the reshaping-inducing molecule was, at least partially, added from outside the vesicle. This is unsuitable, as synthetic cell division should be controlled from “within” the vesicle. In principle, however, the mechanism of division by adsorbed proteins is independent of the direction of the Protein addition. In this study, we set out to computationally identify design parameters that might induce vesicle reshaping and division from within.

## Materials and Methods

**Figure 1:**
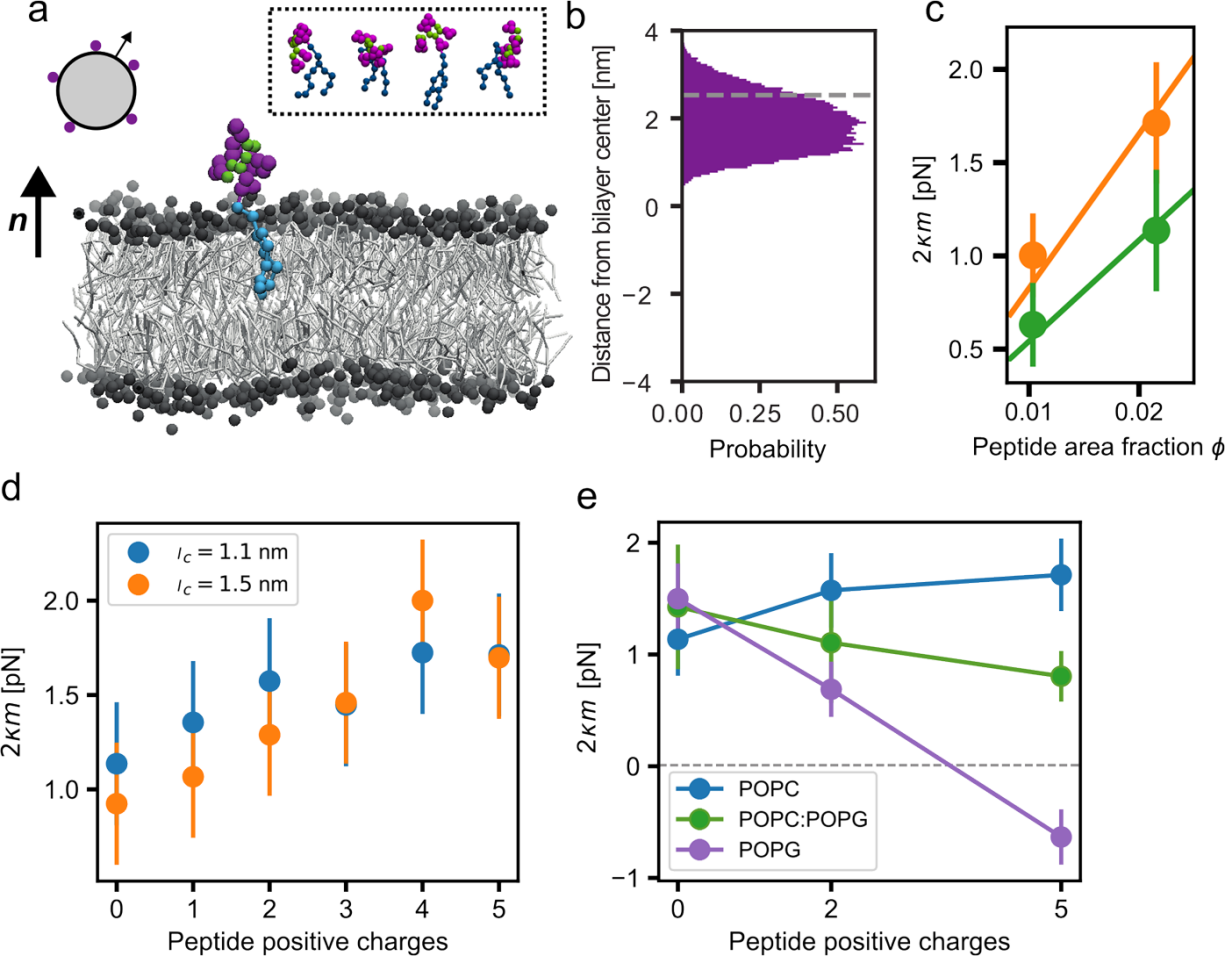
(a) Snapshot of lipid bilayer with peptide:anchor complex (anchor lipid in blue, peptide backbone in green, and His side chains in purple) in a POPC lipid bilayer (gray lipids). The top left sketch shows a vesicle with peptides adsorbed to the outer leaflet. The normal vectors ***n*** at the planar and vesicle bilayer point toward the exterior solution. The inset shows different snapshots of the peptide:anchor complex. (b) Histogram of peptide center-of-mass locations during simulation. The gray line indicates the average position of PO4 beads (membrane–water interface). (c) Parameter 2κ*m* from eq 1 for two peptide area fractions of uncharged peptide (green) and of 5+ charged peptide (orange) in a POPC lipid bilayer. Both fitted lines go through the coordinate (0,0). (d) Parameter 2κ*m* at fixed area fraction  for varying number of peptide charges in a POPC lipid bilayer. Two different values for the electrostatic cutoff *l*_c_ (see inset) are compared. (e) Parameter 2κ*m* at fixed area fraction  for three different membrane compositions (blue, pure POPC; green, 50:50 mixture POPC:POPG; and violet, pure POPG), blue line shows the same data as in panel d for *l*_c_ = 1.1 nm. Error bars indicate the SEM determined from blocking analysis.

We used GROMACS engine version 2018.2 to run MARTINI v2.2 coarse-grained molecular dynamics simulations,^9,10^ using input parameters new-rf and a time step of 20 fs.^11^ We considered two system sizes, 81 or 169 POPC lipids in each leaflet, that equilibrated to bilayer patches of 7.2 and 10.4 nm^2^ on average, as determined by the simulation box size. The membranes were initially constructed with insane.^12^ Lipid bilayers at full hydration (at least 15 water beads, equivalent to 60 water molecules, per lipid) were simulated at 303.15 K and 1 bar with semi-isotropic pressure coupling. Each simulation contained one lipid–peptide complex, which replaced a single POPC lipid. Simulations with charged lipids had the corresponding number of POPG lipids. Counter ions (NA or CL beads) were added to reach overall charge neutrality. The lateral stress profiles *s*(*z*) of the bilayers were calculated with Goetz–Lipowsky decomposition,^13^ implemented in the GROMACS-LS software.^14^ The spontaneous curvature *m* was calculated from the first moment of the stress profile *s*(*z*) as given by^15^
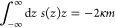
1where *z* is the coordinate perpendicular to the planar bilayer, with the upper leaflet and the exterior solution being located at positive *z*-values. The normal vector is taken to point from the upper leaflet into the exterior solution as in Figure 1a. When the electrostatic cutoff *l*_c_ was varied, both production runs and stress profile calculations were performed with the same *l*_c_ values. The peptide:anchor complex replaced a single lipid in a pre-equilibrated (as determined by the area per lipid) membrane patch. After energy minimization and an additional 200 ps equilibration, the simulation lengths were 1 μs with a sampling rate of 100 ps. The average membrane area covered by the peptide was used to calculate the area fractions ϕ and was estimated from the average end-to-end distance of the peptide, assuming a circular shape of the covered area because the lipid:anchor complex is free to rotate.

**Figure 2:**
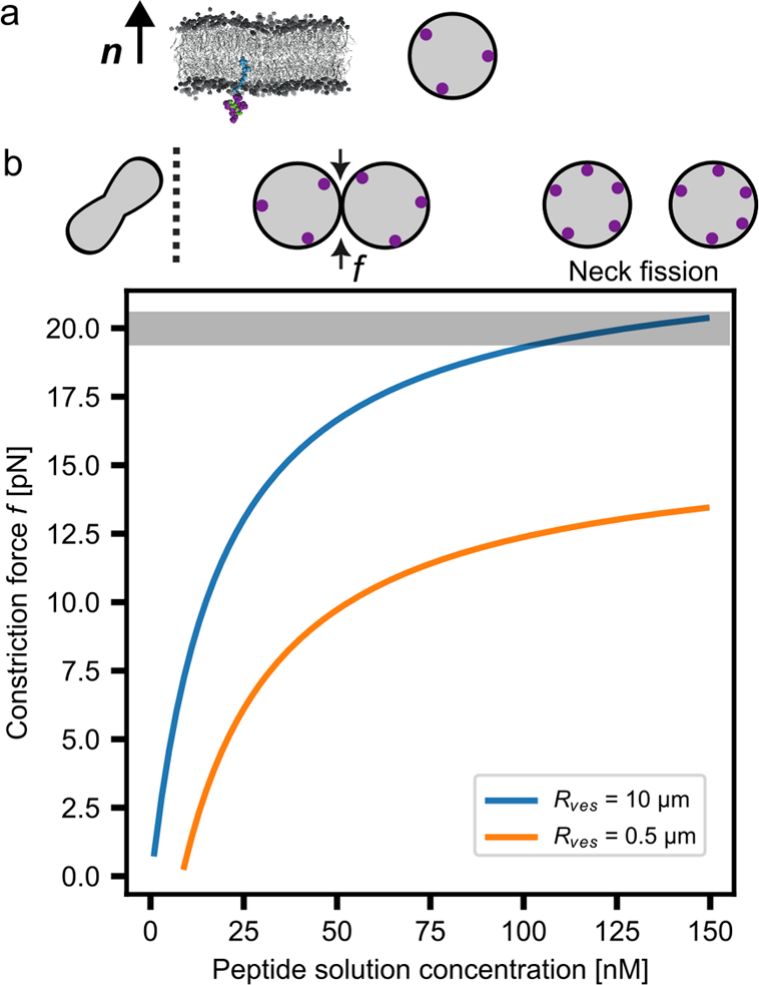
(a) Planar and vesicle bilayer with the peptide:anchor complex bound to the lower and inner leaflets of the bilayer; (b) the top row shows approximate shapes of a deflated vesicle with  that forms a dumbbell with closed neck at , indicated by the dashed line for the vesicle *R*_ves_ = 0.5 μm. Graph shows estimate of constriction force acting on the neck of the dumbbell-shaped cell-sized POPG vesicle with 3 mol % NTA anchor lipids with varying solution concentration of the 5+ charge peptide, compare Figure 1e, that generates constriction force *f* on the closed membrane neck of the dumbbell as computed for two different vesicle radii *R*_ves_ (see inset). The constriction force value for experimentally observed spontaneous neck fission by His-GFP is indicated by the horizontal gray stripe, see ref. (7) for details.

The neck constriction force in Figure 2 was calculated from the constriction force  acting at the membrane neck of a dumbbell vesicle.^8^ Parameters used in Figure 2 are the bending rigidity κ = 25.2*k*_B_*T* and the value 2κ*m* as calculated via eq 1 from the stress profile of pure POPC membranes. The neck curvature  was determined for the two studied values of the vesicle size  for membrane area *A*. The volume-to-area ratio is defined by  for vesicle volume *V*.

## Results and Discussion

For our computational approach, we used coarse-grained molecular dynamics as provided by the MARTINI v2.2 model,^9^ which is well-suited for studying membrane-curvature generation.^16−18^ As a division-inducing biomolecule, we considered six linked beads of the uncharged form of the amino acid histidine (His), which can be viewed as a model for a 6-His peptide. The peptide was strongly anchored to the membrane at its center by a bound interaction—a simple model for strong biomolecular interactions. The choice of the 6-His peptide model is motivated by the well-established NTA-Ni^2+^-His binding that anchors His peptides to a lipid headgroup,^19−21^ together with the observation that the binding affinity exhibits a maximum for a chain length of six histidines.^22^ In addition, His has two charge states that can be switched experimentally by moderate changes in pH, and we will explore the effect of charges later in this work. Initially, we considered a 1-palmitoyl-2-oleoyl-*sn*-glycero-3-phosphocholine (POPC) bilayer, where one POPC molecule was replaced by a peptide–lipid complex. This setup confined the peptide just below the membrane–water interface; no flip-flops between the leaflets or dissociation from the membrane occurred. Thus, the simulated membrane was asymmetric, and we expected the bilayer to exhibit a preferred or spontaneous curvature.^15,18,23,24^ This was indeed observed, as measured from the first moment of the membrane lateral stress profile (Materials and Methods). The stress profile calculation gives the value of 2κ*m*, where κ is the bilayer bending modulus and *m* the spontaneous curvature, at varying peptide densities.^25^ We use the sign convention that the normal vectors of the membrane point toward the exterior water compartment and that bulging of the membrane toward this compartment implies a positive spontaneous curvature. The simulated peptide size (MW = 840 g/mol) is comparable to the lipid size (MW = 760 g/mol). Therefore, these molecules effectively integrate a voluminous lipid headgroup with relatively thin tails, reminiscent of conical lipids such as glycolipids. Previous experiments conducted to estimate the local curvature radius generated by asymmetrically distributed glycolipid GM1 (MW 1547 g/mol) *m*_0_^*–* 1^ resulted in values between 7 and 8 nm.^18^ This value should be compared to our simulation result of 3.8 ± 0.3 nm obtained in this work (green fitted line in Figure 1c). This shows that the bulky peptide:anchors studied here are comparable in their curvature-generation ability to cone-shaped lipids.^23^ Compared to lipids, our peptide system has the benefit of being able to explore the large chemical diversity of peptides that bind to the lipid anchors and provide a “plug-and-play” modification to the membrane because experimentally peptide synthesis is much more accessible than lipid synthesis. Still, the possible sequence space of peptides in experiments is large. Therefore, we chose a computational approach to understand general design elements that would help to guide an experimental approach. This approach allows us to fairly quickly (roughly 3-week simulation on 16 CPU cores per condition) determine the spontaneous curvature of a variety of peptide sequences and lipid compositions for experimentally relevant conditions.

We next asked if we could tune the molecular interactions between the peptide and the membrane. The amino acid His exists in two charged states owing to the (de)protonation of the imidazole side chain. To study the effect of the electrical charges, we increased the number of positively charged His residues and observed that the generated spontaneous curvature increased with the number of charges (Figure 1c, blue points in Figure 1d). For technical reasons, the calculation of the stress profiles requires a fixed electrostatic cutoff length that we initially set to the MARTINI standard value of 1.1 nm, corresponding to strongly screened electrostatic interactions. As there are few mobile charges at the membrane-water interface where the peptide is absorbed, our simulations might underestimate the magnitude of the electrostatic effects. Larger cutoff lengths were studied before, at large computational expense.^14,26,27^ Indeed, setting the cutoff length to 1.5 nm in both our simulation and stress profile calculation led to slightly different results (orange trace in Figure 1d). However, the observed differences between the two cutoff values are small compared to the statistical uncertainty and we did not consider the variation of the electrostatic cutoff length further. Thus, even if the values reported here might underestimate the magnitude of the electrostatic effects, we established an additional design parameter, the peptide charge, to tune the spontaneous curvature of the membrane in this system.

Returning to our initial goal of realizing cell division from within, we saw that increasing the peptide charge increases the tendency of the membrane to bend away from the anchored peptide, the opposite direction needed to realize the division of a vesicle. We reasoned that providing attractive interactions between the peptide and bilayer that locally reduces the area per lipid provides the desired membrane curvature. As there is a large variety of charged lipids, we explored electrostatic attraction between the cationic peptide (5+ HIS) and anionic lipid headgroups. Indeed, including 50% negatively charged 1-palmitoyl-2-oleoyl-*sn*-glycero-3-phosphatidylglycerol (POPG) lipid leads to a decreasing spontaneous curvature which decreases with increasing peptide charge (Figure 1e). In addition, the asymmetric ionic conditions of the two leaflets might contribute to the calculated total spontaneous curvature,^8,28,29^ but we did not try to disentangle these two effects further. Next, we considered a pure POPG membrane as this should maximize the effect that favors bending toward the adsorbed peptide. As anticipated, the sign of the spontaneous curvature at the maximum peptide charge density was reversed (Figure 1e). We remind the reader that this would induce positive spontaneous curvature if the peptide was added to the inner bilayer leaflet. It is important to note that here, we compare the value of 2κ*m* between different lipid compositions. It is likely that the value of the bending rigidity, κ, varies between these systems. Indeed, the lipid charge has been shown to rigidify lipid bilayers.^30^ That said, variation of the overall sign of spontaneous curvature is robust against these changes as the bending rigidity is always positive. Thus, the combination of the POPG membrane and the 5+ charged peptide is predicted to induce synthetic cell division if the peptide is synthesized and adsorbed at the inner membrane leaflet.

Finally, we studied a lipid vesicle with peptide adsorbed to the inner leaflet and estimated the peptide concentration necessary to induce division (Figure 2a). We assumed the recently experimentally determined binding constant (*K*_d_ = 18.5 nM) of 6H-FITC, which represents a 6-His-long peptide tagged with a fluorophore at the N-terminal-binding 3% NTA-anchor-lipid concentration.^31^ We considered two differently sized lipid vesicles with radii *R*_ves_ = 0.5 and 10 μm that form a prolate shaped vesicle with a reduced volume to area ratio of  as shown in Figure 2b. Theory and experimental studies show that such a vesicle deforms into a dumbbell vesicle when the spontaneous curvature exceeds .^7,8,32^ Given these previous studies, we are able to predict the shape changes of a lipid vesicle used as a synthetic cell chassis from the simulations reported here. In the studied conditions, with the peptide adsorbed to the negatively charged inner membrane leaflet, the concentrations predicted to induce a dumbbell shape are 8.5 and 0.3 nM, for the two values of *R*_ves_. Once established, the neck of the dumbbell might be stable for many hours in experiments.^7,32^ However, when the surface coverage of the peptide would be increased further, an increasing constriction force acts on the membrane neck, leading to rapid fission.^7,8^ We calculated the constriction force that is generated at the membrane neck (Figure 2b).^8^ We found that roughly 150 nM solution concentration is sufficient to induce a constriction force of up to 20 pN, quite comparable to the constriction force as obtained by binding His-GFP to giant unilamellar vesicles (GUVs).^7^ In order to determine the constriction force from the experimental data, it is necessary to analyze the GUV morphologies in a systematic and quantitative manner. Notably, the anchor concentration of 3 mol % and small size of the peptide as studied here ensure that peptide–peptide interaction on the membrane can be neglected, and the adsorbed peptide is in the dilute regime. Because the constriction force depends on the size of the dumbbell vesicles, a smaller vesicle might not fission readily (orange trace in Figure 2b). The latter effect that should be considered if these predictions are to be verified experimentally. Taken together, these results suggest that even dilute peptide solution concentration are sufficient to generate membrane neck constriction forces that lead to complete neck fission of cell-sized vesicles.

## Conclusion

This study elucidates peptide–lipid interactions and their role in synthetic cell division using the MARTINI coarse-grained model. We have established the conditions for which peptide charge, membrane composition, and electrostatic interactions are predicted to generate constriction forces that lead to the synthesis of cell division from within. The limitations of the coarsely modeled lipid–peptide binding and peptide models without a secondary structure should be mentioned: it might very well be that the 6-His peptide used here is not ideal, but a different sequence induces division more effectively. For example, switching the charge state of 6-His by pH might interfere with the anchoring strength to the membrane.^31^ Here, a combination of uncharged 6-His and other cationic amino acids might be suitable. In addition, it might be possible that providing slightly longer charged peptides could relax the requirement of pure PG membranes to induce division. Our study indicates the general design rules that a division-inducing peptide should follow. This peptide can be rather short, should have an opposite charge to the membrane lipids, and must bind strongly to a lipid headgroup. These features distinguish our work from previous studies of amphipathic helices that can also generate both positive and negative curvatures by inserting into lipid membranes at varying insertion depths.^33^ The anchor lipid largely suppresses the effect of varying insertion depth and therefore allows one to independently tune the electrostatic interactions, potentially narrowing down the sequence space to a few charged amino acids. The relatively small sequence space is amenable to experimental screening or directed evolution approaches. A suitable experimental screening strategy is the addition of a peptide to the outside of a deflated vesicle and to vary the peptide sequence. In this case, generation of inward buds and their fission indicates that the same peptide bound to the inner leaflet of the vesicle will lead to division of a dumbbell vesicle into two daughter vesicles. Ideally, this would then be combined with peptide synthesis from coding DNA within the synthetic cell, an already established module.^2,4,34^ As the synthesis proceeds, it will first induce the shape change to a dumbbell and, with increasing peptide concentration over time, induce division. Alternatively, the synthesis reaction could be coupled with error-prone DNA replication that would enable a type of directed evolution experiment. For both approaches, the design principles described here will be helpful.
